# Autoclosing Excision Design for Dermatofibrosarcoma Protuberans in Slow Mohs Surgery

**DOI:** 10.1002/ccr3.73472

**Published:** 2026-09-15

**Authors:** Elise Lupon, Ioana Ivan, Olivier Camuzard, Silvia Gandolfi, Dimitri Gangloff

**Affiliations:** ^1^ Department of Plastic and Reconstructive Surgery, Institut Universitaire Locomoteur et du Sport University Côte d'Azur, Pasteur 2 Hospital Nice France; ^2^ Université Côte d'Azur, CNRS LP2M Nice France; ^3^ Department of Plastic and Reconstructive Surgery Institut Universitaire Du Cancer Toulouse Oncopole Toulouse France

**Keywords:** dermatology, inguinal lymph node dissection, melanoma, oncology, surgery

## Abstract

In DFSP treated with slow Mohs surgery, first‐stage planning is critical given delayed histologic results. The pinch test allows intraoperative assessment of skin mobility, enabling margin tailoring beyond the standard 1–1.3 cm when laxity permits, while preserving primary closure. This integrates oncologic and reconstructive planning in a single surgical step.

## Key Clinical Message

In DFSP treated with slow Mohs surgery, planning excision guided by the pinch test allows margin tailoring to individual skin laxity, optimizing the likelihood of negative margins at first excision while preserving primary closure.

**Clinical Question:** How can surgical planning optimize margin management in DFSP treated with slow Mohs surgery?

**Answer:** For dermatofibrosarcoma protuberans (DFSP), European guidelines recommend margins of 1–1.3 cm with slow Mohs surgery, versus up to 3 cm with conventional histology [1]. Because histologic results are available only 24–48 h later, first‐stage planning is critical. The pinch test can be used intraoperatively to assess skin mobility and determine the maximal excision compatible with primary closure increasing the likelihood of negative margins at first excision. By ensuring comprehensive margin control, Mohs surgery may reduce the need for adjuvant radiotherapy, which remains indicated for positive or unresectable margins [2]. This approach is particularly applicable on the trunk and extremities (Video 1).

**VIDEO 1:** Pinch test–guided autoclosing excision design in slow Mohs surgery for dermatofibrosarcoma protuberans. Intraoperative demonstration of the pinch test used to plan an autoclosing excision during dermatofibrosarcoma protuberans resection treated with slow Mohs surgery. Skin mobility is assessed by manual approximation of the surrounding tissues to determine the maximal excision that still allows primary closure. In this approach, margins are not defined by a fixed distance, but by the amount of tissue that can be safely removed while maintaining the possibility of delayed primary closure after histologic margin confirmation. The corresponding margin in centimeters is nevertheless systematically recorded in the operative report. Video content can be viewed at https://onlinelibrary.wiley.com/doi/10.1002/ccr3.73472.

## Author Contributions

**Elise Lupon:** conceptualization, data curation, formal analysis, visualization, writing – original draft, methodology, investigation, writing – review and editing, funding acquisition, resources. **Ioana Ivan:** data curation, writing – review and editing, software. **Olivier Camuzard:** writing – review and editing, supervision, resources. **Silvia Gandolfi:** writing – review and editing, supervision, resources. **Dimitri Gangloff:** resources, validation, supervision, project administration, writing – review and editing, visualization.

## Funding

The authors have nothing to report.

## Consent

Written informed consent was obtained from the patient for publication of this case and the accompanying images and video.

## Conflicts of Interest

The authors declare no conflicts of interest.

## Data Availability

The data that support the findings of this study are available on request from the corresponding author. The data are not publicly available due to privacy or ethical restrictions.
